# Demographic characteristics of blood and blood components transfusion recipients and pattern of blood utilization in a tertiary health institution in southern Nigeria

**DOI:** 10.1186/s12878-018-0112-5

**Published:** 2018-07-31

**Authors:** Henshaw Uchechi Okoroiwu, Ifeyinwa Maryann Okafor

**Affiliations:** Haematology Unit, Department of Medical Laboratory Science, University of Calabar, Calabar, Nigeria

**Keywords:** Transfusion, Blood utilization, Blood usage, Whole blood, International classification of disease (ICD)

## Abstract

**Background:**

An insight into the utilization pattern helps in future planning of blood drive. This study was conducted to describe the demographic characteristics of the transfusion recipients and pattern of blood and blood product utilization in Nigeria.

**Methods:**

Blood bank registers of University of Calabar Teaching Hospital (UCTH) Calabar were analysed for a 12 month period. Number of blood units requested, number of units issued, Cross-match to transfusion ratio (C/T), age, gender, blood group, blood components received, patients ward and clinical diagnosis were computed. Diagnoses were grouped into broad categories according to the disease headings of International Classification of Diseases (ICD-10).

**Results:**

Majority of the 2336 transfusion recipients studied were females (69.09%) and are in the reproductive age group; 15–49 years (75.23%). The median age of the recipients was 35 years (range, 0–89). Most of the recipients (*n* = 1636; 70.04%) received whole blood transfusion. Majority (94.46%) of the cross–matched units were issued giving C/T ratio of 1.06. The common blood group type was O Rhesus positive (62.63%). Obstetrics and Gynecology had the highest blood requisition (41.40%). The majority of the patients were diagnosed with conditions related to pregnancy and childbirth (38.70%), conditions originating in prenatal period (14.38%). The age range of 25–54 years had the highest blood transfusion requests (*n* = 501; 51.07%), of these, females were majority (*n* = 390;77.84%).

**Conclusions:**

Our study recorded mostly young patients who received mostly whole blood. Most of the patients in the reproductive age group received transfusion for pregnancy and child-birth related cases.

## Background

Blood transfusion plays important role in medical and surgical practice [1]. In order to achieve these, critical review and continuous evaluation of the use of blood and its components becomes essential [2]. These entails studying the pattern of blood components use, the clinical conditions and wards requiring blood transfusion, the risks associated with blood transfusion and the demographic characteristics of the blood transfusion recipients in a population. Evaluation of blood requisition and utilization is essential in assessing the present and future demands for blood and avoiding unnecessary requests and transfusions [3]. Despite the development of national blood service policy [4], most medical facilities in Nigeria still find it difficult in establishing viable and efficient blood banking system [5]. On this premise, it becomes necessary to ensure judicious utilization of this scarce commodity. Data on blood utilization is helpful in resource limited settings in which there is always competing needs for scarce resources [6]. Information on blood utilization will assist in establishing clinical practice guidelines, strategizing on new donor recruitment, streamlining resources for the therapeutic benefit of the patient [3, 7] and conducting cost effective analysis [8]. Various studies have shown variable distribution in demand for blood and its components [9, 10], but none has looked into the variability in the blood use based on diseased classification (1CD-10) as well as blood group type of the recipients. Hence, this study sets off to establish local use pattern of blood and blood product to aid in effective management of patient need.

## Methods

### Study setting

This study was conducted in the blood bank unit of the Hematology Department of University of Calabar Teaching Hospital, Calabar, Cross River State, Nigeria which happens to be the only tertiary institution in the state.

### Study design

This study employed retrospective analysis of blood transfusion recipients’ data covering all blood and blood components transfused within the period from March 2016 to February, 2017.

### Data collection

Data were collected retrospectively from the register of the blood bank for the 12 months period from March 2016 to February 2017 and covered all blood and blood components recorded in the blood bank during this period. Cross-match and issue registers were accessed to retrieve the required information such as gender, age, blood group, product requested, ward and clinical diagnosis.

### Statistical analysis

The data collected were analyzed using SPSS version 20 software (Armonk, NY. IBM Corp.). Frequency and percentages were used to summarize categorical demographic and clinical variables.

## Result

A total of 2473 units were requested within the study period consisting of 1770 whole blood, 468 packed cells and 235 plasma. About 94.46% (2336) of the cross matched requests were issued consisting of 1636 (70.04%) whole blood, 467 packed cells (19.99%) and 233 (9.97%) plasma resulting in a cross match to transfusion (C/T) ratio of 1.06 (Table 1).

**Table 1:** Frequency of blood transfusion requests and dispatch based on blood products

Blood component	Units cross matched		Units issued		C/T Ratio
	*N*	(%)	*N*	(%)	
Whole blood	1770	(71.57)	1636	(70.04)	1.08
Packed cell	468	(18.93)	467	(19.99)	1.00
Plasma	235	(9.50)	233	(9.97)	1.01
Total	2473	(100.00)	2336	(100.00)	1.06

*C/T ration* cross-match to transfusion ratio, *%* percentage, *N* absolute number

Most transfusion recipients were female (1614; 69.09%) of whom 475 (29.43%) were in the reproductive age group (15–49 years). Approximately 20% of the transfusion recipients were under the age of 15 while 7% were at least 65 years (Table 2).

**Table 2:** Distribution of age groups of blood transfusion recipients according to age and gender

Age range (years)	Recipient		Males		Females	
	*N*	(%)	*N*	(%)	*N*	(%)
0–14	197	(20.08)	106	(58.81)	91	(46.19)
15–24	123	(12.54)	38	(30.89)	85	(69.11)
25–54	501	(51.07)	111	(22.16)	390	(77.84)
55–64	91	(9.28)	52	(57.14)	39	(42.86)
65+	69	(7.03)	43	(62.32)	26	(37.68)
Not recorded	1355		372		912	
Total	2336		722		1614	

*N* absolute number of subjects, *%* percentage

Table percentages, exclude non recorded values

The most common blood group type observed in the blood/blood component recipients was O Rhesus Positive (1463; 62.63%) while the least was AB Rhesus Negative (0; 0%) (Table 3).

**Table 3:** The distribution of ABO blood groups of blood transfusion recipients according to gender

Blood group	Recipient		Males		Females	
	*N*	(%)	*N*	(%)	*N*	(%)
A^+^	463	(19.82)	159	(34.34)	304	(65.66)
A^−^	47	(2.01)	16	(34.04)	31	(65.96)
AB^+^	4	(0.17)	2	(50.00)	2	(50.00)
AB^−^	0	(0.00)	0	(0.00)	0	(0.00)
B^+^	274	(11.73)	73	(26.64)	201	(73.36)
B^−^	19	(0.81)	11	(57.89)	8	(42.11)
O^+^	1463	(62.63)	442	(30.21)	1021	(69.79)
O^−^	66	(2.83)	19	(28.79)	47	(71.21)
Total	2336	(100.00)	722	(30.91)	1614	(69.09)

*N* absolute number of subjects, *%* percentage

The top six broad 1CD-10 diagnosis categories accounting for most blood transfusion recipients were pregnancy and child birth (*n* = 904; 38.70%), condition originating in perinatal period (*n* = 336; 14.38%), diseases of the genitourinary system (*n* = 184; 7.88%), diseases of the blood and blood forming organs (*n* = 182; 7.79%), Neoplasm (*n* = 156; 6.68%) and injury and poison (*n* = 102; 4. 37%) (Table 4).

**Table 4:** Distribution of blood transfusion recipients based on the broad ICD-10 diagnosis categories

Diagnostic category (ICD-10 code)	Recipients	
	*N*	(%)
Infectious and parasitic diseases	78	(3.34)
Neoplasms	156	(6.68)
Disease of blood & blood forming organs	182	(7.79)
Endocrine, nutritional & metabolic diseases	26	(1.11)
Mental and behavioral disorders	0	(0.00)
Diseases of the nervous system	8	(0.34)
Eye and adnexa diseases	6	(026)
Ear and adnexa diseases	2	(0.09)
Circulatory system diseases	76	(3.25)
Respiratory system diseases	22	(0.94)
Digestive system diseases	92	(3.94)
Skin and subcutaneous tissue diseases	14	(0.60)
Musculoskeletal system & connective tissue diseases	44	(1.88)
Genitourinary system diseases	184	(7.88)
Pregnancy, childbirth & puerperium	904	(38.70)
Condition originating in the perinatal period	336	(14.38)
Congenital malformations	34	(1.46)
Symptoms, signs and abnormal clinical and laboratory findings	62	(2.65)
Injury and poison	102	(4.37)
External causes of morbidity	8	(0.34)
Total	2336	(100.00)

*ICD* International classification of diseases

*n* absolute number, *%* percentage

Obstetrics and Gynecology ward had the highest (967; 41.40%) blood recipients while Eye and Dental/MFU had the least (5; 0.21%) (Table 5).

**Table 5:** Distribution of blood transfusion recipients based on clinical wards

Ward	Recipients	
	*N*	(%)
Medical	378	(16.18)
Orthopedic	114	(4.88)
Cardiothoracic	12	(0.15)
Surgical	198	(8.48)
ENT	18	(0.78)
Eye	5	(0.21)
O & G	967	(41.40)
Pediatrics	22	(0.94)
SCBC	55	(2.35)
ICU	21	(0.90)
CHER	101	(4.32)
Casualty	375	(16.05)
HDCU	65	(2.78)
Dental/MFU	5	(0.21)
Total	2336	(100.00)

*ENT* Ear, Nose and Throat, *O* and *G* Obstetrics and Gynecology, *SCBU* Special care baby unit, *ICU* intensive care unit, *CHER* children Emergency Room, *HDCU* Haematology Day Care unit, *MFU* Maxillofacial unit

A category of the transfusion recipients based on the four broad category showed that more blood requisition in Obstetrics and gynecology (*n* = 967; 41.40%) while the least was pediatrics (*n* = 178; 7.62) (Table 6).

**Table 6:** Distribution of blood transfusion recipients based on broad sections of the hospital

Broad Hospital section	Recipient	
	*N*	(%)
Pediatrics	178	7.62
Surgery	317	13.57
O & G	967	41.40
Medicine	874	37.41

Further stratification of blood components showed that whole blood was utilized more (*n* = 947; 57.89%) in obstetrics and gynecology, while packed cells and plasma were utilized more (*n* = 374; 80.09% and *n* = 188; 80.69%, respectively) in medicine (Table 7).

**Table 7:** Distribution of blood components based on broad sections of the hospital

Broad Hospital section	Blood component		
	Whole blood (%)	Packed cell (%)	Plasma (%)
Pediatrics	82 (5.01)	71 (15.20)	25 (10.73)
Surgery	295 (18.03)	8 (1.71)	14 (6.01)
O & G	947 (57.89)	14 (3.00)	6 (2.57)
Medicine	312 (19.09)	374 (80.09)	188 (80.69)

## Discussion

This study provided information on the pattern of blood and blood components utilization and demographic characteristics of blood transfusion recipients in University of Calabar Teaching Hospital, Nigeria.

This study comprised much of younger cohort of transfusion recipients. This observation is similar to the report of a study in Zimbabwe [6] but in contrast with studies reported from developed countries in which majority of the transfusion recipients were above the age of 60 years [8, 10, 11]. This low median age reflects the age trend of the Nigerian population which comprised mainly of young people with only 3.12% being above the age of 65 years [12]. The life expectancy at birth for Nigeria is currently estimated at 54 years whereas the global population average is 70 years [13]. Developed countries are mainly characterized by ageing population owing to higher mean life expectancies.

This study recorded greater number of female transfusion recipients. Blood transfusion recipients in sub-Saharan Africa are mostly children in malaria endemic areas, and women of childbearing age due to complications of labour [14, 15]. Women, especially the childbearing age group (15–49 years) received the majority of the blood and blood components transfused. This observation is in consonance with findings in other countries in sub-Saharan Africa where women receive more blood for pregnancy-related complications consequent to intra-partum and post-partum hemorrhage [15, 16]. In contrast, studies from developed countries reported that more men than women receive blood transfusion [8, 17]. This may be attributed to advanced health care services which reduced the associated complications of child bearing requiring transfusion [6].

Majority of the transfusion recipients in this study (71.57%) received whole blood transfusion. This is consistent with earlier study in Jigawa Nigeria [18] which reported 87.3%. This is a reflection of common practice of requesting for whole blood in resource limited settings owing to non-availability of facilities to practice component separation. In standard practice, whole blood is only issued for transfusion following cases of massive hemorrhages and exchange transfusion.

This study recorded an average C/T ration of 1.06 which is an indication of effective and efficient utilization of blood and blood products. This finding is similar to 0.9 reported in Ibadan Nigeria [19] but lower than 2.2 reported in Ibadan, Nigeria [20]. The observed differences may be due to the varying levels of availability of blood and indications for blood transfusion as judged by the requesting physician [19, 20].

The top six diagnoses for which patients received blood transfusion in this study were pregnancy and childbirth, conditions originating in perinatal period, diseases of the genitourinary system, diseases of blood and blood forming organs, neoplasm and injury and poison. This finding is similar to earlier report in Zimbabwe [6] with conditions originating in perinatal period being replaced by infections and parasitic diseases. However, other studies in Nigeria did not classify diagnosis according to ICD-10 making direct comparisons with the findings of this study impossible. Studies from non-African decent reported neoplasms, injury and poison, digestive system diseases and circulatory system diseases as the main diagnosis associated with transfusion [21, 22]. This strongly portrays that blood utilization pattern vary significantly within regions and according to practice as well as patients clinical findings. More so, diseases burden, level of organization and advancement of healthcare in the different settings also contribute to the significant differences in blood utilization [6].

The obstetrics and Gynecology ward had the highest blood requisition (41.40%). This finding is similar to previous reports [18, 23]. This observation may be related to the fact that most obstetric and gynecological events may be bleeding related. Peri-partum hemorrhage has been reported as common indication for blood transfusion in obstetric events [24]. More so, the fact that majority of the subjects were females of child bearing age contributed to this outlying peak. Further classification of the blood transfusion recipients based on the four broad classification showed obstetrics still had the highest requisition (41.40%) while pediatrics had the least (7.62%). This finding is similar to previous report by Musa et al. [9] who reported 42.79 and 11.67%, respectively in a study in Zaria, Nigeria. However, their study made 6 broad classification; splitting some areas of internal medicine into trauma and emergency.

Whole blood was mostly used by obstetrics and gynecology while packed cells and plasma were mostly used in medicine. Ideally, blood is effectively used by processing it into components such as red cell concentrates, platelet concentrates, plasma (fresh frozen plasma) and cryoprecipitate [25], but lack of facility for component separation as in our institution makes such difficult. Published guidelines based on “expert opinion” recommends transfusion of plasma for the following clinical indications: active bleeding in the setting of multiple coagulation factor deficiencies (massive transfusions, disseminated intravascular coagulation); emergency reversal of warfarin in patients with active bleeding in settings where prothrombin complex concentration with adequate level of factor VII is not available; and for use as replacement when performing plasma exchange [26–30]. Specifically, these are seen in burns [31, 32], oncology [33], obstetric events [34–36], and more. Packed red cells are indicated in conditions requiring prevention of anemia related tissue hypoxia [37]. Indications for packed cell transfusion include acute sickle cell crisis (for prevention of stroke), acute blood loss greater than 1500 mL or 30% of blood volume [38].

The distribution of ABO blood groups among blood recipients in this study is consistent with that reported in donor population in Nigeria [39]. Acute blood shortage of specific group is a common event in Nigerian hospitals, hence making an understanding of the distribution of blood groups among transfusion recipients important. This information is essential in planning for blood drive as well as distribution of blood and blood components; subsequently ensuring that patients receive blood matching their ABO blood group and Rhesus type [6]. Of the 33 blood group systems representing over 300 antigens as listed by International Society of Blood Transfusion, ABO and Rhesus blood groups are the first two most clinically important blood groups [40, 41].

This study has a number of potential limitations. However, this study was carried out in a major tertiary health institution in Calabar, Nigeria, but the extrapolation of the entire findings of this study to Nigeria may need some caution. More so, this study made the assumption that issued blood units were transfused.

## Conclusion

This study recorded greater number of women receiving blood and blood product transfusion for conditions associated with pregnancy and childbirth. The blood recipients were mostly young patients of reproductive age group. We found that the most indications for blood transfusion based on ICD-10 were pregnancy and child birth, conditions originating in perinatal period, diseases of genitourinary system and diseases of blood and blood forming organs. Whole blood was the major blood component recorded in this study. This shows a lag in healthcare improvement and unnecessary waste of blood. Although this study was based on a single blood bank, the findings provided an insight into the characteristics of blood transfusion recipients and as well aid in future planning of better blood and blood product utilization.

## Abbreviations

C/T: Cross match to transfusion ratio
CHER: Children Emergency Room
ENT: Ear, Nose and Throat
HDCU: Haematology Day Care Unit
HREC: Health Research Ethical Committee
ICD: International Classification of Disease
ICU: Intensive Care Unit
MFU: Maxillofacial Unit
O & G: Obstetrics and Gynecology
SCBC: Special Care Baby Unit
UCTH: University of Calabar Teaching Hospital

## References

[1] Outta AB (2006). Transfusion practice clinical aspects and application. Blood banking and transfusion.

[2] Lim YA, Lee WG, Chow SR (2004). A study of blood usage by diagnosis in a Korean University hospital. Vox Sang.

[3] Zimmermann R, Buscher M, Linhardt C (1997). A survey of blood component use in a German University hospital. Transfusion.

[4] National Blood Transfusion Service (2006). Federal Ministry of Health [Nigeria]. The National Blood Policy. Abuja: National Blood Transfusion Service, Federal Ministry of Health.

[5] Okoroiwu HU, Okafor IM, Asemota EA, Okpokam DC. Seroprevalence of transfusion-transmissible infections (HBV, HCV, syphilis and HIV) among prospective blood donors in a tertiary health care facility in Calabar, Nigeria; an eleven years evaluation. BMC Public Health. 2018;18:645. 10.1186/s12889-018-5555-x 10.1186/s12889-018-5555-x PMC5964952 29788937

[6] Nyashadzaishe M, Star K, Oliver H, Brian EF, Isaac K (2015). Profile of blood and blood component transfusion recipients in Zimbabwe. Blood Transfus.

[7] Biggin K, Warner P, Prescott R (2010). A review of methods used in comprehensive, descriptive studies that relate to red blood cell transfusion to clinical data. Transfusion.

[8] Borkent-Raven BA, Janssen MP, Van der Poel CL (2010). The PROTON study; profiles of blood products transfusion recipients in the Netherlands. Vox Sang.

[9] Musa AU, Ndakotsu MA, Hassan A, Klish A, Kwaifa IK (2014). Pattern of blood transfusion request and utilization at a Nigerian University teaching hospital. Sahel Med J.

[10] Wells AW, Llewelyn CA, Casbard A (2009). The EASTER study; indications for transfusion and estimates of transfusion recipient numbers in hospitals supplied by the national blood service. Transfus Med.

[11] Vamvakas EC, Taswell HF (1994). Epidemiology of blood transfusion. Transfusion.

[12] CIA. The world factbook. Available from: https://www.cia.gov/library/publications/the-world-factbook/geos/ni.html. Accessed in 27 Aug 2017.

[13] World Health Organization. Life Expectancy. Available from: http://www.who.int/gho/mortality_burden_disease/life_tables/en/. Accessed in 27 Aug 2017.

[14] Schneider WH (2013). History of blood transfusion in sub-saharan Africa. Transfus Med Rev.

[15] Bugge HF, Karlsen NC, Oydna E, Rake MM, Wexels M, Bendabenda J (2013). A study of blood transfusion services at a district hospital in Malawi. Vox Sang.

[16] Natukunda B, Schonewille H, Smit-sibinga CT (2010). Assessment of the clinical transfusion practice of a regional referral hospital in Uganda. Transfus Med.

[17] Geiβler RG, Franz D, Buddendick H, Krakowitzky P, Bunzemeier H, Roeder N (2012). Retrospective analysis of the blood component utilization in a university hospital of maximum medical care. Transfus Med Hemother.

[18] Aliyu I, Michael G, Ibrahim H, Ibrahim ZF, Aliyu G, Isaiah AT (2017). Blood transfusion request pattern in a medical Centre in northwestern Nigeria. Glob J Transfus Med.

[19] Fasola FA, Kotila RA, Shokunbi WA (2009). Audit of red cell units supply of a busy hospital blood bank in Nigeria. Niger J Clin Pract.

[20] Ebose EM, Osalumese IC (2009). Blood shortage cituation: an audit of red blood cell order and pattern of utilization. Afr J Biotechnol.

[21] Malthoulin-Pelissier S, Salmi LR, Verret C, Demoures B (2000). Blood transfusion in a random sample of hospitals in France. Transfusion.

[22] Goncalez TT, Sabino EC, Capuani L, Capuani L, Liu J, Wright DJ (2012). Blood transfusion utilization and recipient survival at hospital das clinicas in Sao Paulo, Brazil. Transfusion.

[23] Kagu MB, Ahmed SG, Askira BH (2007). Utilization of blood transfusion service in north eastern Nigeria. Highland Med Res J.

[24] Osei EN, Odoi AT, Owusu-Ofori S, Allan JP (2013). Appropriateness of blood product transfusion in the obstetrics and gynecology (O&G) department of a tertiary hospital in West Africa. Transfus Med.

[25] World Health Organization (WHO). Blood safety and availability. 2017. Available at: http://www.who.int/news-room/fact-sheets/detail/blood-safety-and-availability. Accessed 25 June 2018.

[26] O’Shaughnessy DF, Atterbury C, Bolton MP (2004). Guidelines for the use of fresh-frozen plasma, cryoprecipitate and cryosupernatant. Br J Hematol.

[27] Roback JD, Caldwell S, Carson J (2010). Evidence-based practice guideline for plasma transfusion. Transfusion.

[28] Szczepiorkowski ZM, Winters JL, Bandarenko N (2010). Guidelines on the use of therapeutic apheresis in clinical practice-evidence-based approach from the apheresis Applcation Committee of the American Society for apheresis. J Clin Apher.

[29] Ferraris VA, Brown JR, Society of Thoracic Surgeons Task Force (2011). 2011 update to the Society of Thoracic Surgeons and Society of Cardiovascular Anesthesiologists blood conservation clinical practice guidelines. Ann Thorac Surg.

[30] Yang L, Stanworth S, Hoewell S, Doree C, Murphy M (2012). Is fresh froze plasma clinically effective? An update of a systematic review of randomized controlled trial. Transfusion.

[31] Lu RP, Lin F, Ortiz-Pujols SM, Adams SD, Whinna HC, Carns BA, Key NS (2013). Blood utilization in patients with burn injury and associated with clinical outcomes. Transfusion.

[32] Jones LM, Brown N, Philips G, Blay BA, Bhatti P, Miller SF, Coffey R (2015). Burn resuscitation with fresh froze plasma: 5 years of experience with the west Penn formula. Austn J Emerg Crit Care Med.

[33] Federici AB, Vanelli C, Arrigoni L (2012). Transfusion issues in cancer patients. Thromb Res.

[34] Cata JP, Gottumkkala V (2014). Blood transfusion practices in cancer surgery. Indian J Anaesth.

[35] Gupte SC, Patel PN (2014). Blood transfusion practice in obstetrics and gynecology: impact of educational programs to create awareness for judicious use of blood components. Indan J Hematol Blood Transfus.

[36] Chhabra S, Namgyal A (2014). Rationale use of blood and its components in obstetric-gynecological practice. J Mahatma Ghandi Inst Med Sci.

[37] Muller MM, Geisen C, Seifried E (2015). Transfusion of packed red cells. Dtsch Arztebl Int.

[38] Klein HG, Spahn DR, Carso JL (2007). Red blood cell transfusion in clinical practice. Lancet.

[39] Mathew EE, Godwin NB (2008). Distribution of ABO and rhesus-D blood groups in the Benin area of Niger Delta: implication for general blood transfusion. Asian J Transfus Sci.

[40] Logdberg L, Reid ME, Lamont RE, Zelinski T (2005). Human blood group genes 2004: chromosomal locations and cloning strategies. Transfus Med Rev.

[41] Logdberg L, Reid ME, Zelinski T (2011). Human blood group genes 2010: chromosomal locations and cloning strategies revisited. Transfus Med Rev.

